# Supplementary material for: Phosphate Capture Enhancement Using Designed Iron Oxide-Based Nanostructures
Source: Nanomaterials (Basel). 2023 Feb 1;13(3):587. doi: 10.3390/nano13030587 (PMC9921849; doi:10.3390/nano13030587)
Supplement: Supplementary file 1 [file nanomaterials-13-00587-s001.zip › nanomaterials-2149308-supplementary.pdf]

nanomaterials MDPI

Supplementary Materials

Phosphate Capture Enhancement Using Designed Iron Oxide-Based Nanostructures

Paula Duenas Ramirez [1], Chaedong Lee [2], Rebecca Fedderwitz [3], Antonia R. Clavijo [4], Débora P. P. Barbosa [4], Maxime Julliot [1], Joana Vaz-Ramos [1,5], Dominique Begin [5], Stéphane Le Calvé [5], Ariane Zaloszyc [5], Philippe Choquet [6], Maria A. G. Soler [4], Damien Mertz [1], Peter Kofinas [3], Yuanzhe Piao [2,7] and Sylvie Begin-Colin [1,*]

[1] Institut de Physique et Chimie des Matériaux de Strasbourg, UMR 7504, University of Strasbourg, CNRS, 67034 Strasbourg, France
[2] Graduate School of Convergence Science and Technology, Seoul National University, 145 Gwanggyo-ro, Yeongtong-gu, Suwon-Si 16229, Gyeonggi-do, Republic of Korea
[3] Department of Chemical and Biomolecular Engineering, University of Maryland, 4418 Stadium Dr., College Park, MD 20740, USA
[4] Institute of Physics, University of Brasilia, Brasilia 70910900, Brazil
[5] Institut de Chimie et Procédés pour l'Energie, l'Environnement et la Santé (ICPEES), UMR-7515 CNRS-Université de Strasbourg, 25 rue Becquerel, 67087 Strasbourg, France
[6] Laboratoire des Sciences de l'Ingénieur, de l'Informatique et de l'Imagerie (ICube)—CNRS/University of Strasbourg, UMR 7357 Preclinical Imaging Lab, Imaging Dpt, Hôpitaux Universitaires de Strasbourg, 67098 Strasbourg, France
[7] Advanced Institutes of Convergence Technology, 145 Gwanggyo-ro, Yeongtong-gu, Suwon-si 16229, Gyeonggi-do, Republic of Korea
* Correspondence: sylvie.begin@ipcms.unistra.fr or sylvie.begin@unistra.fr; Tel.: +33-388-107-192

Optimization of the synthesis of iron oxide Nanostructures

Reproducibility of RSN synthesis. The protocol of Gerber *et al.*[1] was first reproduced by using the same reactants (iron precursor flask: Alfa-Aesar 2) and under the same conditions. Under such experimental conditions, Gerber et al. [1] obtained RSN with a mean diameter of 250 nm and a mean nanograin size of 25 nm as measured by TEM. The crystallite size determined by XRD profile matching is 15.5 nm and the saturation magnetization is 78 emu·g^{-1} (Table S1). These new RSN were characterized with SEM, TEM, FTIR spectroscopy, X-Ray diffraction and magnetic measurements. Magnetic measurements were performed in using a Superconducting Quantum Interference Device (SQUID) magnetometer (Quantum Design MPMS-XL 5). Magnetization curves as a function of a magnetic field (M(H) curves) were measured at 300 K. Magnetization saturation (M_S) was measured from hysteresis recorded at 300 K and was determined after removing the mass of organic ligands accordingly to TGA experiments.

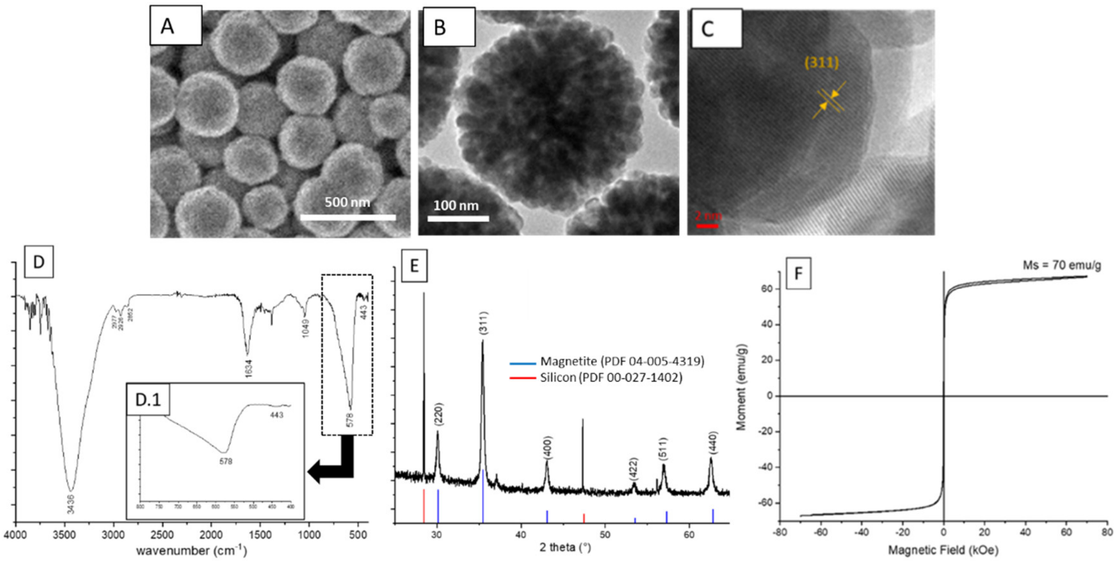

Figure S1. A) SEM, B) TEM and C) HRTEM images of RSN. D) FITR spectrum of RSN with in insert D1) Zoom of FTIR spectrum in the range 800-400 cm^{-1} corresponding to Fe-O bands characteristic here of a slightly oxidized magnetite. E) XRD pattern and F) Magnetization curves of RSN at 300 K.

The SEM image (Figure S1A) is representative of the so synthesized RSN. Large nanoclusters with a mean size of 157 ± 42 nm composed of small nanograins are observed. The **TEM image** (Figure S1B) confirms their aggregate morphology. In the high resolution TEM image (Figure S1.C), the lattice fringe corresponds to the (311) reflection of the cubic spinel iron oxide. This image shows also the parallel lattice fringes between consecutive nanograins confirming the oriented aggregation of grains.

Concerning the magnetite and/or maghemite composition of these RSN, **the XRD pattern** (Figure S1E) displays the characteristic diffraction peaks of an iron oxide spinel structure. No peaks of other phases (hematite, wüstite) are visible suggesting a good purity of the nanoclusters. The crystallite size (determined by profile matching) from the XRD pattern is of 20.2 nm. The lattice parameter determined by profile matching is 8.399 ± 0.02 Å and is slightly higher than that of magnetite (JCPDS file 39-1346). This may be explained by the oriented aggregation of grains which may induce local strains that impact the lattice parameter value [2].

The **FTIR spectroscopy** allows to discriminate between magnetite and maghemite phases (Figure S1.D). In fact, in the range 800-400 cm^{-1} corresponding to Fe-O bands maghemite (the oxidized phase of magnetite) displays broad bands. The resolution of these bands depends on the structural order of vacancy sites in maghemite. By contrast, magnetite presents only a well-defined band at 570-590 cm^{-1} [3–6]. In the Figure S1D1, IR spectrum of RSN presents a single well-defined band at 578 cm^{-1} and a shoulder is noticed at higher wavenumbers. This suggests a main composition of magnetite but slightly oxidized. Moreover, at 3435 cm^{-1}, the FTIR spectrum exhibits a characteristic band of the O-H groups of adsorbed water molecules (water asymmetric stretching). Other bands attributed to water molecules are observed at 1610 cm^{-1} and 1110 cm^{-1} corresponding to the absorbed water antisymmetric stretching and O-H stretching. Small bands around 2911 cm^{-1} are observed that would correspond to CH_2 and CH antisymmetric and symmetric stretchings. Depending on the washing step, a band at 1100 cm^{-1} is also clearly visible, which is related to C=O bond from ethylene glycol (EG). The presence of the band related to EG shows that the washings were insufficient to remove all organic

reactants and products. Several washing steps have been performed but it was always difficult to remove completely EG.

About the magnetization curve at 300 K (Figure S1F), no hysteresis loop was observed confirming the superparamagnetic behavior of the RSN. The measured magnetization saturation (Ms) is about 70 emu·g^{-1}.

Table S1. Comparison of the characteristics of RSN of Gerber *et al.* [2] and the RSN of this work.

		Gerber *et al.* (sample RSN25)	This research
**Size of RSN (nm)**		245 ± 12	157 ± 42
**Size of nanograins (nm)**[3]		25 ±	30 ± 6
**Crystallite size (nm by XRD)**		15.5	20.2 ± 0.2
**Lattice parameter (A)**		8.39	8.39
**Magnetic properties**	**M_S (emu·g^{-1})**	78	70

Iron carbonates formation

Another encountered problem was the random formation of iron carbonates within the final product (Figure S2). Only in the sample with more than 5% of carbonates, two diffraction peaks (104) and (018) related to iron carbonates (PDF 00-029-0696) were identified (Figure S2C).

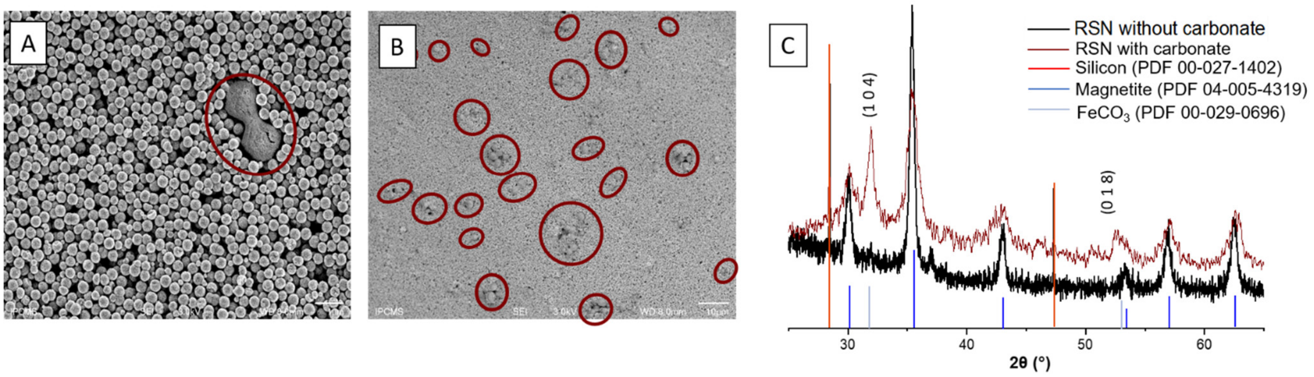

Figure S2. SEM images corresponding to the synthesis of RSN without A) and with B) iron carbonates (brushes). XRD pattern of both RSN batches.

Analysis of $FeCl_3{\cdot}6H_2O$ precursors

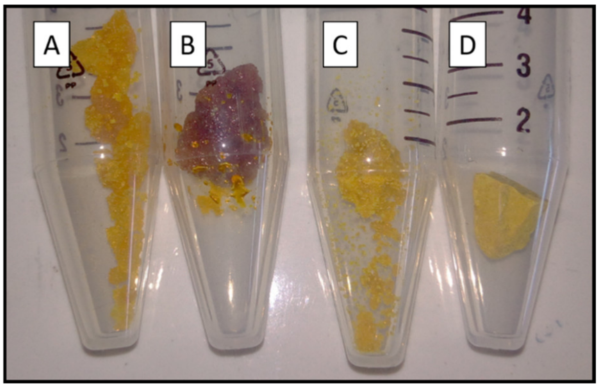

Figure S3. Images of the four flasks containing the different iron precursors: A. Sigma 1, B. Alfa Aesar 1, C. Alfa Aesar 2, and D. Acros Organics 1.

SEM-EDS results

SEM images of the four reactants (Figure S4) show the same morphology: a rough but uniform surface without grains or sheets. Moreover, we do not observe a strong charge of energy in images, which is characteristic of organic and less conductive species.

The results of EDS elementary analysis (Figure S4) show the presence of some element traces. In addition to iron, chloride and oxygen, the reactants also contain:

- Sigma and Alfa Aesar 2: aluminum, magnesium and silicium.
- Alfa Aesar 1: carbon, aluminum and silicium.
- Acros Organics: aluminum and silicium.

Additionally, the certificates of analysis provided by the different producers were checked. In this case,

- Sigma: traces of arsenic, copper, lead, zinc, phosphates and sulfate.
- Alfa Aesar reactant (1 and 2) do not contain impurities.
- Acros organics: some traces of heavy metals, arsenic and sulfates.

The comparison of our analysis with the furnished data showed some differences. Such differences could be an accidental pollution of the flask by other users (the flasks are in common use and are shared with other teams), or, less probably, a non-indicated pollution by the producer.

However, all reactants contain similar impurities and thus that does not allow explaining the observed differencies in characteristics of RSN.

Iron amount determined by relaxometry measurements

To calculate the iron amount, a given amount of iron chloride hexahydrate in water was dissolved and a determined amount of nitric acid (65 %) is added. Then, the mixture is diluted to reach a final concentration of 2 % in nitric acid. A relaxometry measurement is performed in a Brucker Minispec Instrument (60 MHz, 1.41 T), which measures the relaxation time of iron cations. Thanks to a calibration curve, which associates the iron concentration to the relaxation time T1, the iron concentration of the sample is determined. The theoretical iron weight and the experimental one deduced by relaxometry measurements are presented in Table S2.

Table S2. Amount of iron in the different iron precursors determined by relaxometry measurements.

Iron Precursor	Sigma 1	Alfa Aesar 1	Alfa Aesar 2	Acros Organics 1
Iron $mass_{calculated}$ (mg)	1	1	1	1
Iron $mass_{experimental}$ (mg)	0.89	0.93	0.94	0.93
%	-1.5	-6.8	-5.9	-7.1

These measurements evidence that the iron amount in Sigma 1 is 10.5 % lower than the expected amount and for Alfa Aesar 1, 2 and Acros Organics, the iron deficit is of 6.8, 5.9 and 7.1 %, respectively. All batches of iron chloride (III) hexahydrate contain less iron than expected and it suggests a higher water content. These results would be in agreement with different hydration rate of precursors. So, if the reactant is more hydrated, the diameter of RSN decreases. This observation agrees with the experiment of Cao et al. [7] who observed that when water is added, the size of nanoclusters decreases.

From SEM-EDS results (Figure S4), atomic ratios between some important elements: Fe and Cl (from $FeCl_3$) and Fe and O (from H_2O) were determined. In theory, an atomic Fe/Cl ratio of 0.33 and Fe/O of 0.165 should be observed. In general, all the samples contain an atomic ratio Fe/Cl slightly higher that the theoretical one (Sigma: 0.35, AA1: 0.36, AA2: 0.38 and Acros 0.44). It may either be due to an increase in Fe or a decrease in Cl. As the Fe amount is lower than expected (Table S2), the increase of this ratio suggests that the amount of chlorine decreases (and maybe replaced by another molecule). The size of RSN seems to decrease with the increase of this ratio.

In addition, the ratio Fe/O is much higher than the expected one: Sigma: 0.33, AA1: 0.30, AA2: 0.38 and Acros: 0.35. It corresponds to an average of 50 % more than expected. It could suggest either a higher amount of iron or a lower amount of molecules providing oxygen such as water or possibly OH. Indeed, under hydration, $FeCl_3{\cdot}6H_2O$ should transform into $FeCl_2(H_2O)_4$ and finally to $Fe(H_2O)_6$ (in water). In this evolution, we can note than the ratio Fe/O decreases. Therefore, from all these results, one may conclude that the iron precursors are hydrated. The water for hydration of hexahydrate iron chloride may come from the air if the flaks of the reactant were not carefully stored.

The difficulty of this investigation is how to evaluate the degree of hydrolysis in the sample (if possible) and also to be sure that we have only hydration and not hydrolysis.

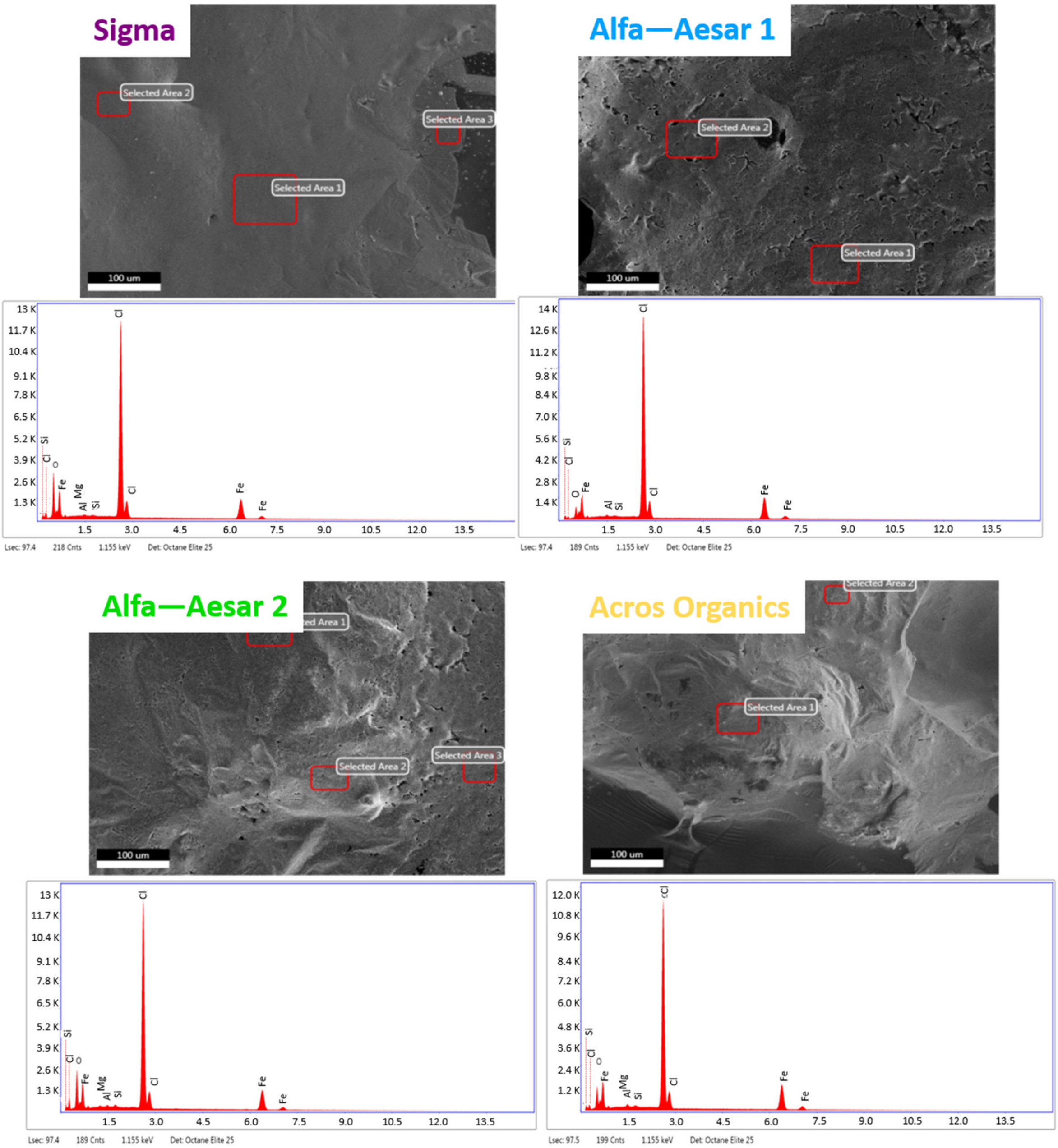

Sample	Fe/Cl	Fe/O
Sigma 1	0.35	0.33
Alfa Aesar 1	0.36	0.30
Alfa Aesar 2	0.38	0.38
Acros Organics 1	0.44	0.37

Figure S4. SEM images and EDX graphs corresponding to the iron precursors. Table summarizing the atomic ratio of Fe/Cl and Fe/O.

Hydration of $FeCl_3.6H_2O$

To better understand what happened with iron III chloride precursors, its hydrolysis has been studied in detail. Hexahydrate ferric chloride has a crystallographic structure conformed to a ferric ion surrounded by two Cl^- ions and four

water molecules under the form of trans-$[FeCl_2(OH_2)_4]^+$. In the crystal, these ions and water molecules will be positioned by hydrogen bonds and electrostatic forces (Figure S5). The crystals have a space group C2/m with a=11.89±0.02, b=7.05±0.01, c=5.99±0.01 Å. The unit cell has two units type $[FeCl_2(OH_2)_4]Cl{\cdot}2H_2O$.[8]

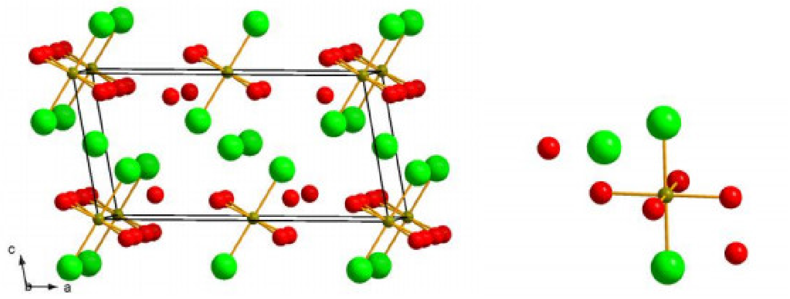

Figure S5. (Left) crystal structure of $FeCl_3{\cdot}6H_2O$ and (right) asymmetric unit. Iron is represented in brown, water in red and chlorine in green[9].

However, the hydration of iron chloride may occur with the time. Gradually, the Cl ligands are replaced by H_2O, so, the possible reactions are:

$$FeCl_3{\cdot}6H_2O \rightarrow [FeCl_2(OH_2)_4]^{+}+H_2O \rightarrow [FeCl(OH_2)_5]^{2+} + Cl^- \text{ (partial hydrolysis)}$$

$$[FeCl(OH_2)_5]^{2+}+ H_2O \rightarrow [Fe(OH_2)_6]^{3+} + Cl^- \text{ (complete hydrolysis)}$$

In agreement with Jolivet *et al.*, during the hydration, chlorine ligands are replaced by aqueous ligands to provide hexa-coordinated cations in an octahedral environment [10].

The reaction in water is : $FeCl_x{\cdot}nH_2O \rightarrow [Fe(H_2O)]_6 + zCl$

A hydrolysis may also occur [11], the charger transfer in the bond H_2O-M makes the O-H bond weaker and the acidity of the aquo-ligand increases and finally, the deprotonation of this ligand becomes possible, and several couples of acidic and basic species are present.

$$[Fe(H_2O)]_6 + H_2O \rightarrow [Fe(OH)_h(H_2O)_{6-h}]^{(z-h)+} + hH^+$$

Jolivet [10] proposed a model to determine h, directly linked to the hydrolysis of iron:

$$h = \left[\frac{1}{1 + 0.014\,pH}\right] \times [1.36z - N(0.236 - 0.038pH) - \frac{2.261 - 0.02pH - \chi m}{\sqrt{\chi m}}]$$

Here, χm is the Muliken electronegativity (1.72 for iron), and z is the formal charge (+3), N is the coordination number (6). The difference in hydrolysis is not due to the initial concentration [11].

To calculate the h of the hydrolysis of iron, the different iron precursors were dissolved in water to obtain a concentration of 0.1 M and then pH was measured.

Table S3. $pH_{measured}$, $h_{calculated}$ and main species for the different iron precursors at 0.1 M (in theory).

Reactant	$pH_{measured}$	$h_{calculated}$	Main species
**Sigma1**	1.97	2.66	$(Fe(OH)_{2.66}(H_2O)_{3.34})^{0.34+}$
**AA1**	1.90	2.64	$(Fe(OH)_{2.64}(H_2O)_{3.36})^{0.36+}$
**AA2**	1.79	2.62	$(Fe(OH)_{2.62}(H_2O)_{3.38})^{0.38+}$
**AO1**	1.84	2.63	$(Fe(OH)_{2.63}(H_2O)_{3.37})^{0.37+}$

Table S3 shows that if the amount of water is high enough, the iron precursors may be hydrolyzed.

XRD, FTIR and TGA analyses of the different iron precursors have been performed to try to identify the different species inside the reactants: $[FeCl_2(OH_2)_4]^+$, $[FeCl(OH_2)_5]^{2+}$ and $[Fe(OH_2)_6]^{3+}$.

<u>*XRD*</u>

XRD patterns (Figure S6A) of the different iron chlorides show only XRD peaks characteristics of $FeCl_3{\cdot}6H_2O$ (PDR 00-033-0645) with similar intensity. No other peaks are visible, suggesting a conservation of the crystallinity of the reactants and the presence of a unique phase. In fact, the partial replacement of Cl by OH during the hydrolysis preserves the structure.

<u>*FTIR spectroscopy*</u>

FTIR spectra (Figure S6B) are quite similar: the bands around 3217 cm^{-1} and 1596 cm^{-1} are attributed to the O-H band linked to the crystalline water inside the structure. The band at 3524 cm^{-1} is related to hydroxyl-metal groups [12]. The band at 1415 cm^{-1} could be attributed to the metal-chlorine bonds [13]. When comparing these samples, the difference in intensity of this last band could confirm the hydrolysis. Surprisingly, the band at 840 cm^{-1} often linked with silicon (i.e. Si-O-Si [14]and Si–N [15]) matches with the silicon traces detected by the SEM-EDS. The triplet between 470 and 780 cm^{-1} is characteristic of an iron oxyhydroxyde (FeOOH). Thus, the FTIR spectra confirm the contamination of the products with silicon and the partial hydration of the precursors.

<u>*TGA curves*</u>

TGA experiments (Figure S6C) were performed to try to quantify the amount of water inside the samples. The four curves are similar to other published TGA curves [16]. $FeCl_3{\cdot}6H_2O$ loses its ligands and finally forms an iron oxide product. The first step at 37 °C corresponds to the melting point of iron chloride (III) hexahydrate, the following ones are related to ligand weight loss and formation of other products: $Fe(OH)Cl_2{\cdot}H_2O$ around 73 °C, $Fe(OH)_2Cl$ above around 150 °C, FeOOH above around 207 °C and finally Fe_2O_3 above 430 °C. Between room temperature and 110 °C, the weakly bonded water molecules are removed (dehydration). The strongly bonded water molecules are removed in a temperature range between 110 and 207 °C. Finally, between 210 and 600 °C, the anhydrite iron chloride is decomposed and HCl and Cl are removed [16]. Nevertheless, TGD (not presented here) helped to observe that transition temperatures are not exactly the same for different precursors. Acros Organics reactant has lower transition temperatures than Alfa Aesar 2, 1 and Sigma 1. According to the previous analysis, $FeCl_3{\cdot}6H_2O$ theoretically contains 59.9 % of water. From TGA curves, the coordinated water was evaluated to be 58.8 %, 58.9 %, 59.31 % and 59.85 % for Sigma 1, AA1, AA2 and Acros Organics 1 samples, respectively. It would correspond to an increase in water amount of 1.8, 1.6, 1.0 and 0.08 % in precursors, respectively. Differences in transition temperatures could be related to the different rates of hydration. Nonetheless, TGA has to be carefully interpreted. In general, the results depend on the sample preparation, and, as previously said, iron chloride is very hygroscopic and tends to easily form aggregates. It can be difficult to obtain similar samples between the preparation and the analysis. In that case, this analysis method cannot allow to determine the amount of water, but clear differences can be noticed.

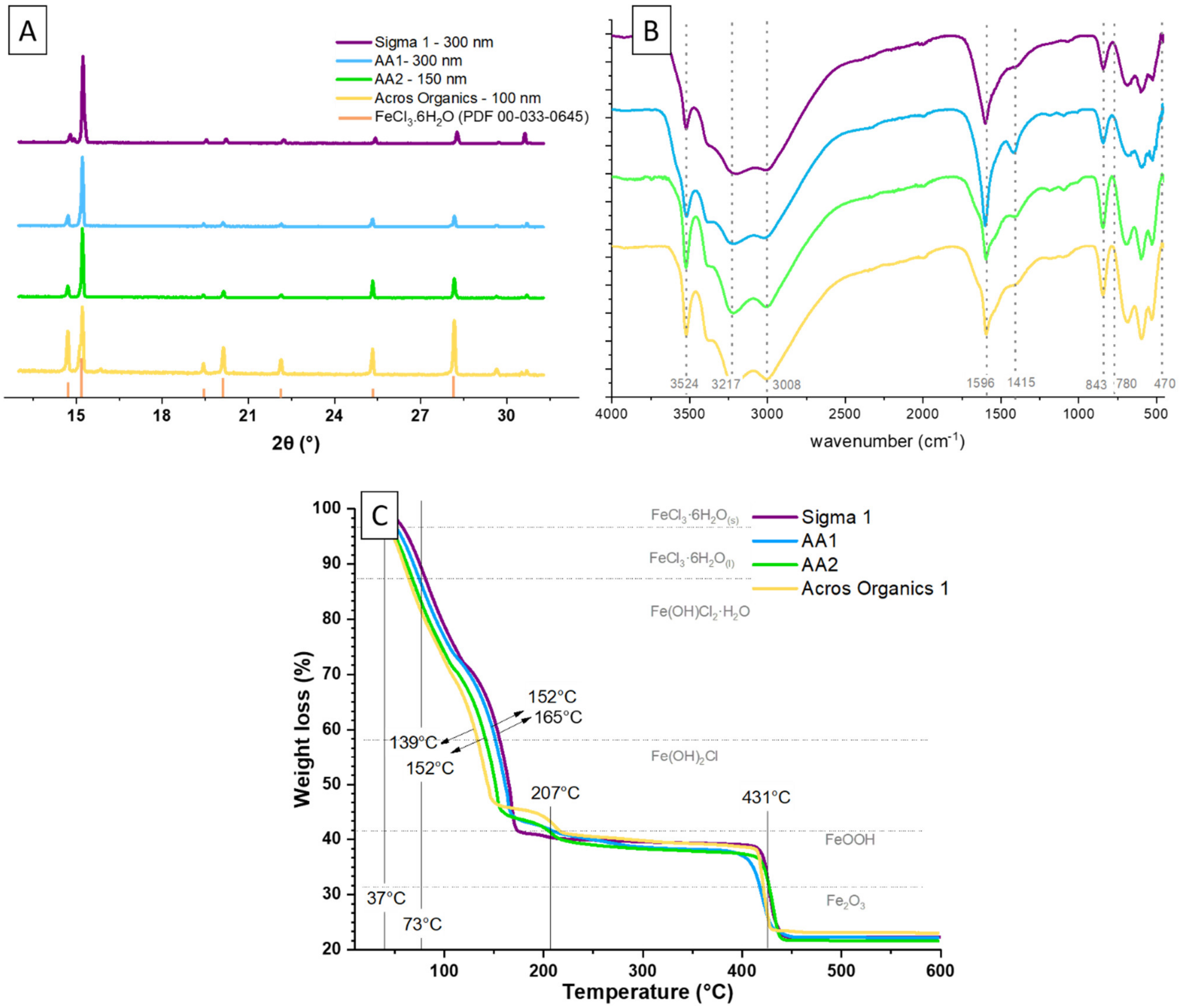

Figure S6. A. XRD patterns under air, B. FTIR spectra and C.TGA curves under air (5°C/min) of the different iron precursors.

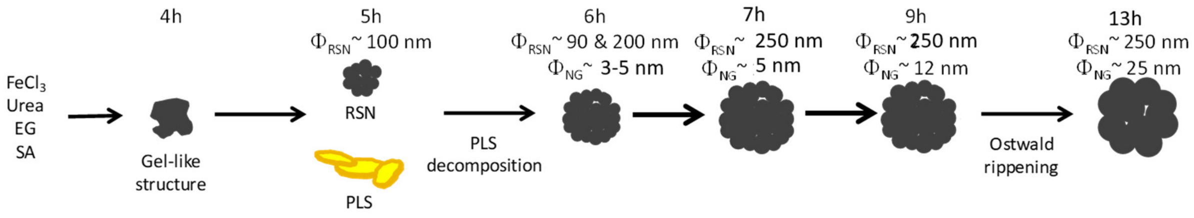

Figure S7. Reminder: Reaction steps of the synthesis of magnetite RSN.[1].

Structural and magnetic characterizations of RSN synthesized with the new iron precursor

To overcome problems linked to hydrolysis of iron reactants, a "new/fresh" flask has been used_and new RSN synthesized. A typical RSN synthesis (3 h of mix and 10.5 h of reaction) leads to the expected raspberry-shaped morphology (Figure S8A). TEM image (Figure S8B) confirms the formation of aggregates of nanograins. These nanoclusters have a diameter around 296 ± 35 nm with nanograins of ca. 25 nm (measured from TEM images). Moreover, the homogeneous contrast in the nanostructure confirms that these objects are not hollow. In the HRTEM image (Figure S8C1), resolved

lattice fringes are observed. The distance between two adjacent planes in a specific direction were determined to be around 2.5 Å, corresponding to the *hkl* planes of the spinel iron oxide (*311*). Selected area electron diffraction (SAED) pattern (Figure S8C2) were taken from the sampling area of Figure S8C1. The SAED pattern shows concentric rings, which evidences the good crystallinity of RSN. Moreover, the distances between the rings and the center of the pattern can all be attributed to a *hkl* plane from the spinel structure, which confirms the crystallization of RSN in a spinel phase. Based on these observations, one can conclude that the RSN are clusters of single crystals with similar crystallographic orientations.

Figure S8D displays the FT-IR spectrum of the RSN sample. As for the previously reported RSN, the characteristic bands of the O-H bond (water) at 3435 $cm^{-1,}$ 1610 cm^{-1} and 1110 cm^{-1} are observed. Small bands at 2911 cm^{-1} corresponds to C-H_2 and C-H bonds and the peak at 1100 cm^{-1} is related to C=O showing the presence of traces of EG on the surface of RSN. Finally, in the zone 800-400 cm^{-1}, the well-defined band at 580 cm^{-1} confirmed the magnetite composition.

Figure S8E represents a typical XRD pattern of RSN. Silicon was used as reference to set and correct the Y-shift due to sample preparation. The diffraction peaks are well-defined and can be indexed to the cubic spinel structure of magnetite or maghemite (magnetite JCPDS file 19-629 and maghemite JCPDS file 39-1346). Rietveld refinement allowed to determine the crystallite size (30 nm) and the lattice parameter (a= 8.401 Å). The crystallite size did not completely correspond to the ones measured from TEM images and it is related, as explained above, to the mechanism of formation of the oriented aggregates. The lattice parameter value higher than that of bulk magnetite is explained also by the presence of strains and defects in such oriented aggregates [2].

Magnetization curve at 300 K between -70 kOe and 70 kOe in Figure S8F confirms their superparamagnetic behavior (no hysteresis) and yields a saturation magnetization value of ca. 90 $emu \cdot g^{-1}$, a value close to that of bulk magnetite (92 $emu \cdot g^{-1}$) [17].

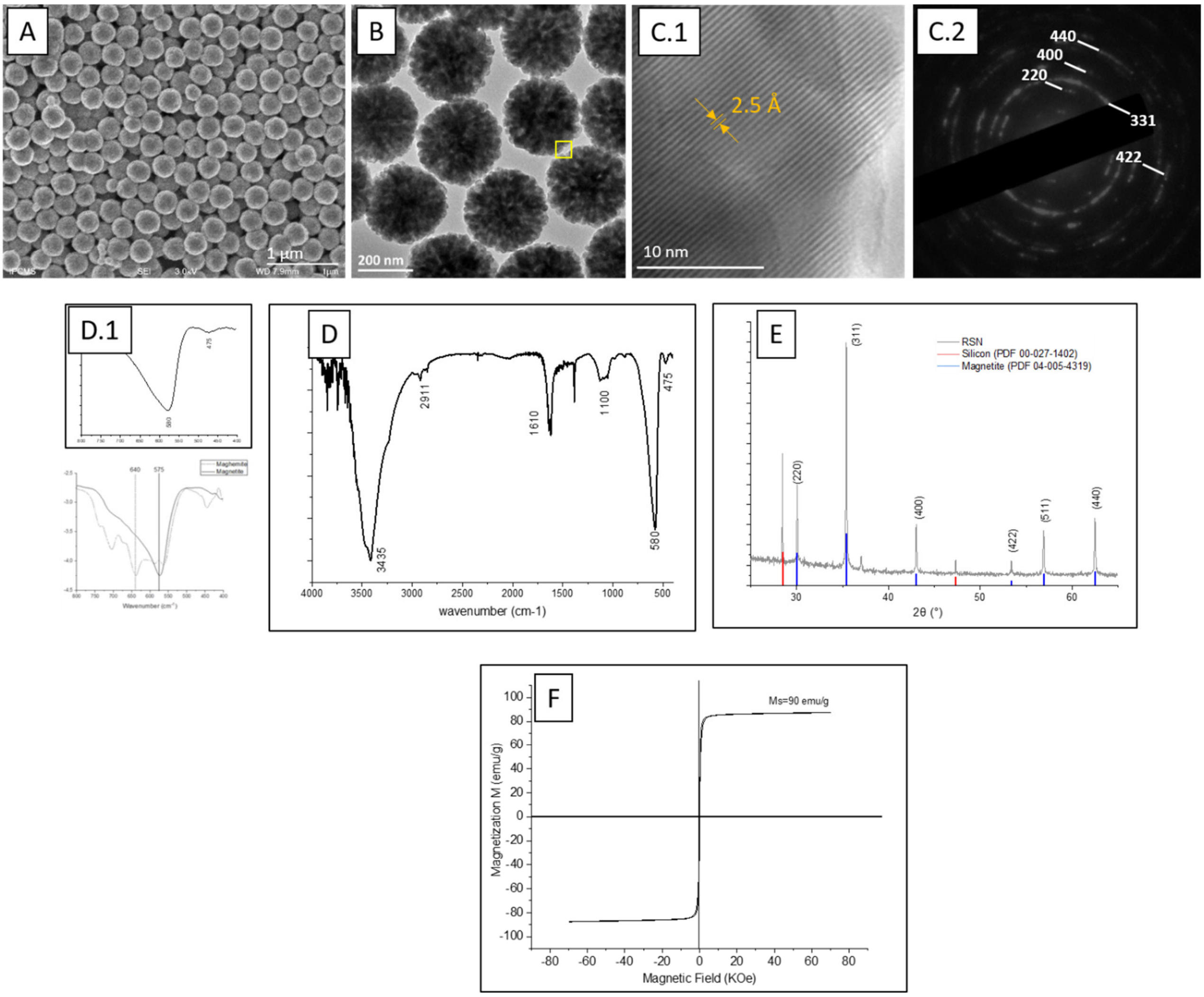

Figure S8. A) SEM, B) TEM and C1) HRTEM images of RSN; C2) SAED pattern; D) XRD pattern. E) FT-IR and F) Magnetization curve at 300K.

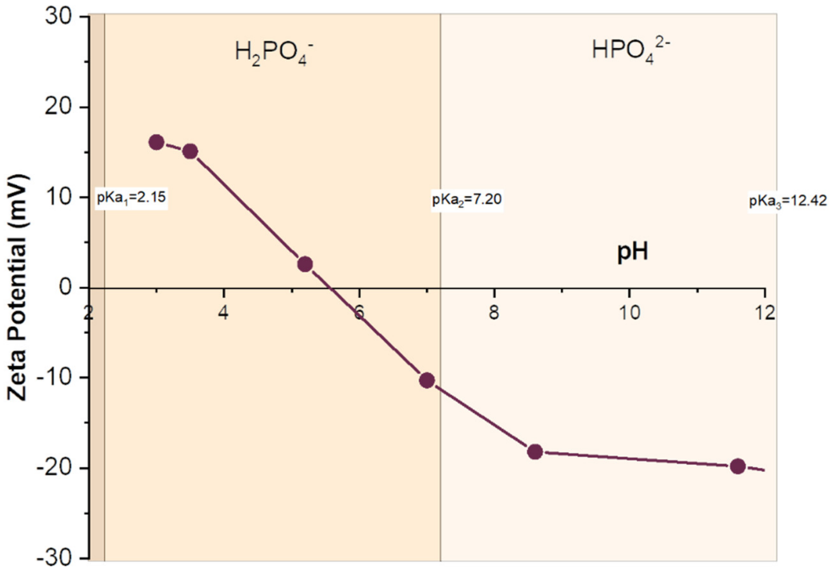

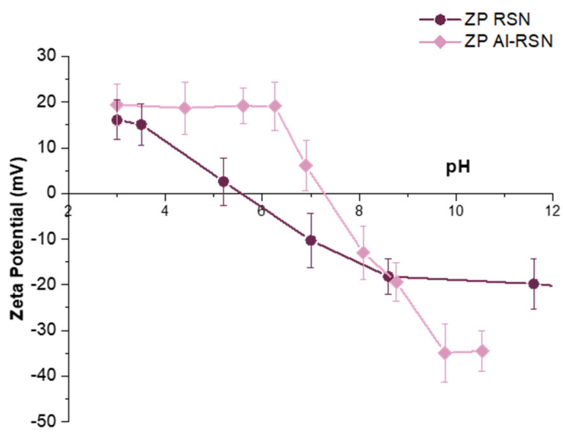

Figure S9. Zeta potential curve of RSN vs pH and phosphate species as a function of pH (top) and Zeta Potential curves of RSN (in violet) and Al-RSN (in pink) as a function of pH (down).

Table S4. Kinetics results of different iron-based materials.

Iron Material	Size	Specific Surface Area ($m^2 \cdot g^{-1}$)	pH	Media	Kinetic model	q_e (P-mg·g^{-1}) and conditions	K_2 (h^{-1})
**Yoon *et al.* Iron oxide NPs**[18]	20 nm	82.2	/	water	Second	4.93<br>T=30 °C<br>Adsorbent :0.6 g·L^{-1}<br>Ce = 2 P-mg·L^{-1}	2.21
**Zeng *et al.* Iron oxide Tailings**[19]	69	48	6-6.8	water	First and second models are not adapted	/	/
**Lalley *et al.* Bayoxide® E33 (goethite based)**[20]	Rod-like structures ca. 500 nm	140	7	Water lake	Second	27.47<br>T=21 °C<br>Adsorbent :5 g·L^{-1}<br>Ce = 140 P-mg·L^{-1}	0.59
**Shahid *et al.* Aggregates of Iron oxide NPs**[21]	Aggregates of NPs of 11.6 nm	75.8	6.8	Water	No data		
**Mezenner *et al.* Iron hydroxide-eggshell**	iron hydroxide-eggshell waste 50–315 µm	/	7	water	Second	1.45<br>T=21 °C<br>Adsorbent : 7.5 g·L^{-1}<br>Ce = 53 P-mg·L^{-1}	1.58
**Cao *et al.* Iron oxide NPs**[22]	Structure of about 160 nm formed by NPs of 80 nm	/	1.62	water	Second	8.20<br>T=25 °C<br>Adsorbent : 2 g·L^{-1}<br>Ce = 10 P-mg·L^{-1}	2.52
**Ajmal *et al.* Iron oxide NPs**[23]	32–55 nm	123	7	water	Second	7.96<br>T=35 °C<br>Adsorbent : / g·L^{-1}<br>Ce = 100 P-mg·L^{-1}	1.2
**Daou et al. Iron oxide NPs**[24]	40 nm	30	No data				
**This study**	300	27	7	water	Second	4.1	5.28

**RSN**						T=20 °C<br>Adsorbent : 1 g·L^{-1}<br>Ce = 50 P-mg·L^{-1}	

Table S5. Kinetics results of different aluminum-iron oxide materials.

Iron Material	Size	Specific Surface Area (m^2·g^{-1})	pH	Media	Kinetic model	q_e (P-mg·g^{-1}) and conditions	K_2 (h^{-1})
**Li** ***et al.*** **AM0**[25]	Goetithe wires around 500 nm	19.1	5.5	Water	Second	0.42<br>T=25 °C<br>Adsorbent : 0.5 g·L^{-1}<br>Ce = 0.5 P-mg·L^{-1}	7.60
**Li** ***et al.*** **AM3**[25]		22.1	5.5	Water	Second	0.44<br>T=25 °C<br>Adsorbent : 0.5 g·L^{-1}<br>Ce = 0.5 P-mg·L^{-1}	6.14
**Li** ***et al.*** **AM6**[25]		26.8	5.5	Water	Second	0.48<br>T=25 °C<br>Adsorbent : 0.5 g·L^{-1}<br>Ce = 0.5 P-mg·L^{-1}	5.16
**Li** ***et al.*** **AM9**[25]		31	5.5	Water	Second	0.51<br>T=25 °C<br>Adsorbent : 0.5 g·L^{-1}<br>Ce = 0.5 P-mg·L^{-1}	5.47
**De Sousa** ***et al.*** **50FeAl**[26]	Iron oxide mi-crosphe-res 0.85 mm	340	7	Water	No data		
**Xu** ***et al.*** **Al-NP**[27]	Iron oxide NPs 14.24 nm	/	<8	Water	Second	30.3<br>T=25 °C<br>Adsorbent : 0.3 g·L^{-1}<br>Ce = 10 P-mg·L^{-1}	10.20
					Second	43.86<br>T=25 °C<br>Adsorbent : 0.3 g·L^{-1}<br>Ce = 20 P-mg·L^{-1}	0.16
					Second	47.62<br>T=25 °C<br>Adsorbent : 0.3 g·L^{-1}<br>Ce = 30 P-mg·L^{-1}	1.08
**This study Al-RSN**	269 nm	40	7	Water	First	9.8<br>T=20 °C<br>Adsorbent : 1 g·L^{-1}<br>Ce = 50 P-mg·L^{-1}	2.04
					Second	10.3<br>T=20 °C<br>Adsorbent : 1 g·L^{-1}<br>Ce = 50 P-mg·L^{-1}	0.37
**This study RSN**	300	27	7	Water	Second	4.1<br>T=20 °C<br>Adsorbent : 1 g·L^{-1}<br>Ce = 50 P-mg·L^{-1}	5.28

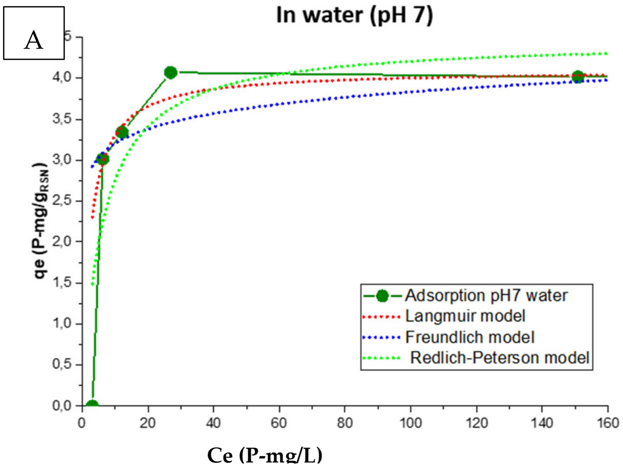

Isotherm model	Parameters	Water (pH 7)	R²
Langmuir	q_m (P-mg/g)<br>K_L(L/mg)	4.1<br>0.42	0.7256
Freundlich	K_f (mg/g)<br>1/n	2.7<br>0.08	0.28481
Redlich-Peterson	K_R (L/mg)<br>a_R (mg-1)<br>g	0.72<br>0.16<br>1	0.7368

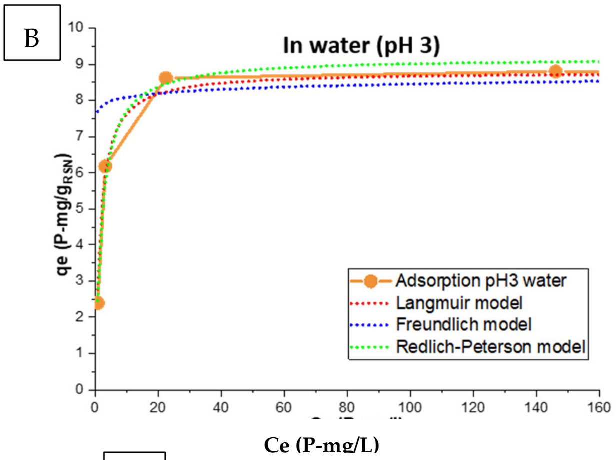

Isotherm model	Parameters	Water (pH 3)	R²
Langmuir	q_m (P-mg/g)<br>K_L(L/mg)	8.8<br>0.66	0.9869
Freundlich	K_f (mg/g)<br>1/n	7.74<br>0.02	0.3647
Redlich-Peterson	K_R (L/mg)<br>a_R (mg-1)<br>g	4.79<br>0.52<br>1	0.99

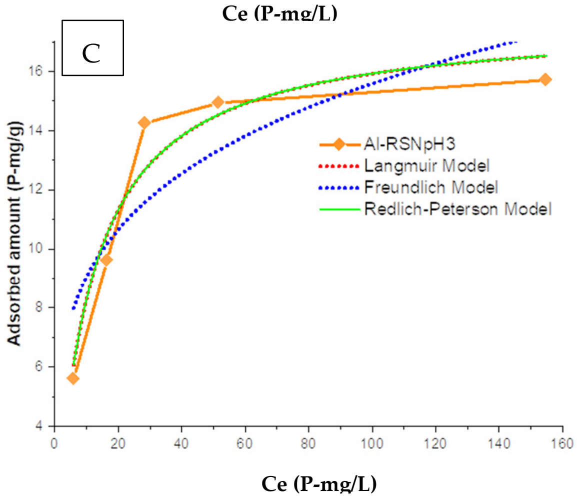

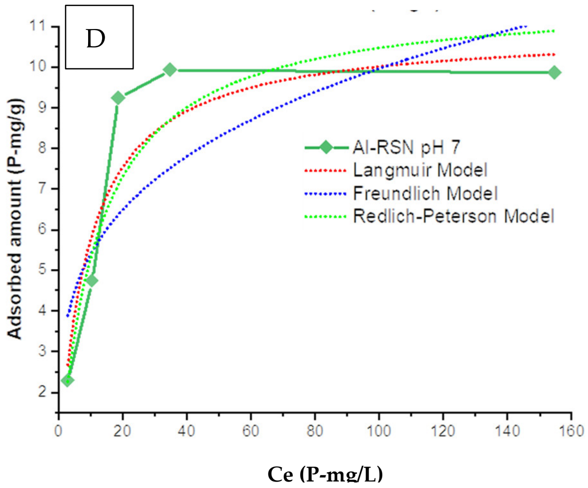

Isotherm model	Parameters	Water (pH 3)	R²
Langmuir	q_m (mg/g)<br>K_L(L/mg)	17.72<br>0.88	0,92231
Freundlich	K_f (mg/g)<br>n	5,2<br>4,2	0.67187
Redlich-Peterson	K_R (L/mg)<br>a_R (mg-1)<br>g	1,57<br>0,088<br>1	0.88346

Isotherm model	Parameters	Water (pH 7)	R²
Langmuir	q_m (mg/g)<br>K_L(L/mg)	10,9<br>0.112	0,75267
Freundlich	K_f (mg/g)<br>n	2,93<br>3,76	0.44681
Redlich-Peterson	K_R (L/mg)<br>a_R (mg-1)<br>g	0,97<br>0,082<br>1	0.76846

Figure S10. Fitting results of the isotherm adsorption curves for RSN A-B) and Al-RSN C-D) (Ce= equilibrium concentration). In red, the Langmuir model, in blue the Freundlich model and in green, the Redlich-Peterson model. Tables summarizes the parameter values corresponding to this fitting.

Table S6. Adsorption isotherm results for different iron oxide-based materials.

Iron Material	Specific Surface Area ($m^2·g^{-1}$)	pH	Media and experimental conditions	Model adsorption	q_m (P-mg·g^{-1})	Adsorption parameters
**Yoon** ***et al.*** **Iron oxide NPs**[18]	82.2	/	Water<br>T= 30 °C<br>t= 24 h<br>Adsorbent : 0.6 g·L^{-1}<br>Ce = 2-20 P-mg·L^{-1}	Redlich-Peterson	5.03	a_R=4.19·10^4 L·mg^{-1}<br>Kr= 1.7·10^5 mg·g^{-1}<br>g=0.88
**Zeng** ***et al.*** **Iron oxide Tailings**[19]	48	6-6.8	Water<br>T= 20-21 °C<br>t= 24 h<br>Adsorbent : 2 g·L^{-1}<br>Ce = 5-150 P-mg·L^{-1}	Freundlich	12.65	K_F= 3.59 L·g^{-1}<br>1/n= 0.19
**Lalley** ***et al.*** **Bayoxide® E33 (goethite based)**[20]	140	7	Lake Water<br>T= 25 °C<br>t= 2 h<br>Adsorbent: 2 g·L^{-1}<br>Ce = 10-100 P-mg·L^{-1}	Langmuir	37.74	K_L= 1.359 L·mg^{-1}<br>R_L< 0.06
**Mezenner** ***et al.*** **Iron hydroxide-eggshell**[28]	/	7	Water<br>T= 25 °C<br>t= 4 h<br>Adsorbent : 0.75 g·L^{-1}<br>Ce = 7-140 P-mg·L^{-1}	Freundlich	10.6	K_F=0.22 mg $L^{-1/n}$ g^{-1} $L^{1/n}$<br>1/n= 0.78
**Cao** ***et al.*** **Iron oxide NPs**[22]	/	1.6	Water<br>T= 25 °C<br>t= 3 weeks<br>Adsorbent: 2 g·L^{-1}<br>Ce = 10-100 P-mg·L^{-1}	Langmuir	18.69	K_L= 0.41 L·mg^{-1}<br>R_L< 0.2
**Ajmal** ***et al.*** **Iron oxide NPs**[23]	123	7	Water<br>T= 25 °C<br>t= 2 h<br>Adsorbent : 2 g·L^{-1}<br>Ce = 200-1000 P-mg·L^{-1}	Freundlich	57.8	K_F 2.07 mg/g<br>1/n= 1.29
**Daou et al. Iron oxide NPs**[24]	30	7	Water<br>T= RT °C<br>t= 24 h<br>Adsorbent : 1 g·L^{-1}<br>Ce = 3-1500 P-mg·L^{-1}	/	1.5	No data
		3		/	5.2	
**This study RSN**	27	7	Water; T= 20 °C<br>t= 24 h<br>Adsorbent : 1 g·L^{-1}<br>Ce = 3.1-154.9 P-mg·L^{-1}	Langmuir	4.1	K_L =0.42 L·mg^{-1}<br>R_L< 0.43
**This study RSN**		7	Dialysis solution	Langmuir	4.1	K_L =0.42 L·mg^{-1}<br>R_L< 0.43
**This study RSN**		3	Water	Langmuir	8.8	K_L =0.66 L·mg^{-1}<br>R_L< 0.33

Table S7. Adsorption isotherm results of different aluminum doped ferrite materials.

Iron Material	pH	Media and experimental conditions	Model adsorption	q_m (P-mg/g)	Parameters
**Li *et al.* AM0**[4]	5.5	Water<br>T= 25 °C<br>t= 24 h<br>Adsorbent : 0.5 g·L^{-1}<br>Ce = 0.07–1.7 P-mg·L^{-1}	Langmuir	1.21	K_L= 5.04 L·mg^{-1}<br>R_L< 0.74
**Li *et al.* AM3**[4]	5.5	Water<br>T= 25 °C<br>t= 24 h<br>Adsorbent : 0.5 g·L^{-1}<br>Ce = 0.07–1.7 P-mg·L^{-1}	Langmuir	1.23	K_L= 6.41 L·mg^{-1}<br>R_L< 0.69
**Li *et al.* AM6**[4]	5.5	Water<br>T= 25 °C<br>t= 24 h<br>Adsorbent : 0.5 g·L^{-1}<br>Ce = 0.07–1.7 P-mg·L^{-1}	Langmuir	1.36	K_L= 6.48 L·mg^{-1}<br>R_L< 0.69
**Li *et al.* AM9**[4]	5.5	Water<br>T= 25 °C<br>t= 24 h<br>Adsorbent : 0.5 g·L^{-1}<br>Ce = 0.07–1.7 P-mg·L^{-1}	Langmuir	1.65	K_L= 4.54 L·mg^{-1}<br>R_L< 0.76
**De Sousa *et al.* 50FeAl**[70]	7	Water<br>T= 30 °C<br>t= 24 h<br>Adsorbent : 7.5 g·L^{-1}<br>Ce = 1–140 P-mg·L^{-1}	Langmuir	8.21	K_L= 1.04 L·mg^{-1}<br>R_L< 0.49
**Xu *et al.* Al-NP**[44]	<8	Water<br>T= 25 °C<br>t= overnight<br>Adsorbent : 0.6 g·L^{-1}<br>Ce = 1-40 P-mg·L^{-1}	Langmuir	102.15	K_L= 1.09 L·mg^{-1}<br>R_L< 0.48
	<8	Water<br>T= 25 °C<br>t= overnight<br>Adsorbent : 1.2 g·L^{-1}<br>Ce = 1-40 P-mg·L^{-1}	Langmuir	81.31	K_L= 1.09 L·mg^{-1}<br>R_L< 0.48

References

1. Gerber, O.; Pichon, B.P.; Ihiawakrim, D.; Florea, I.; Moldovan, S.; Ersen, O.; Begin, D.; Grenèche, J.-M.; Lemonnier, S.; Barraud, E.; et al. Synthesis Engineering of Iron Oxide Raspberry-Shaped Nanostructures. *Nanoscale* **2016**, *9*, 305–313, doi:10.1039/C6NR07567C.
2. Gerber, O.; Pichon, B.P.; Ulhaq, C.; Grenèche, J.-M.; Lefevre, C.; Florea, I.; Ersen, O.; Begin, D.; Lemonnier, S.; Barraud, E.; et al. Low Oxidation State and Enhanced Magnetic Properties Induced by Raspberry Shaped Nanostructures of Iron Oxide. *J. Phys. Chem. C* **2015**, *119*, 24665–24673, doi:10.1021/acs.jpcc.5b08164.
3. Tartaj, P.; Morales, M. P.; Veintemillas-Verdaguer, S.; González-Carreño, T.; Serna, C.J. The Preparation of Magnetic Nanoparticles for Applications in Biomedicine. *J. Phys. D: Appl. Phys.* **2003**, *36*, R182–R197, doi:10.1088/0022-3727/36/13/202.

4. Nakamoto, K. *Infrared and Raman Spectra of Inorganic and Coordination Compounds*; 4th ed.; John Willey and Sons: New York, 1986;
5. Daou, T.J.; Pourroy, G.; Bégin-Colin, S.; Grenèche, J.M.; Ulhaq-Bouillet, C.; Legaré, P.; Bernhardt, P.; Leuvrey, C.; Rogez, G. Hydrothermal Synthesis of Monodisperse Magnetite Nanoparticles. *Chem. Mater.* **2006**, *18*, 4399–4404, doi:10.1021/cm060805r.
6. Baaziz, W.; Pichon, B.P.; Fleutot, S.; Liu, Y.; Lefevre, C.; Greneche, J.-M.; Toumi, M.; Mhiri, T.; Begin-Colin, S. Magnetic Iron Oxide Nanoparticles: Reproducible Tuning of the Size and Nanosized-Dependent Composition, Defects, and Spin Canting. *J. Phys. Chem. C* **2014**, *118*, 3795–3810, doi:10.1021/jp411481p.
7. Cao, S.-W.; Zhu, Y.-J.; Chang, J. Fe_3O_4 Polyhedral Nanoparticles with a High Magnetization Synthesized in Mixed Solvent Ethylene Glycol–Water System. *New J. Chem.* **2008**, *32*, 1526–1530, doi:10.1039/B719436F.
8. Lind, M.D. Crystal Structure of Ferric Chloride Hexahydrate. *J. Chem. Phys.* **1967**, *47*, 990–993, doi:10.1063/1.1712067.
9. Louvain, N.; Fakhry, A.; Bonnet, P.; El-Ghozzi, M.; Guérin, K.; Sougrati, M.-T.; Jumas, J.-C.; Willmann, P. One-Shot versus Stepwise Gas–Solid Synthesis of Iron Trifluoride: Investigation of Pure Molecular F2 Fluorination of Chloride Precursors. *CrystEngComm* **2013**, *15*, 3664–3671, doi:10.1039/C3CE27033E.
10. Jolivet, J.-P.; Henry, M. *De la solution à l'oxyde - Condensation des cations en solution aqueuse. Chimie de surface des oxyde*; EDP Sciences, 1994; ISBN 978-2-7598-0292-0.
11. Perton, F. Architecture de Nanoparticules Hybrides Pour Une Imagerie et/Ou Thérapie Multimodales. University of Strasbourg, 2019.
12. Guzman, A.; Zuazo, I.; Feller, A.; Olindo, R.; Sievers, C.; Lercher, J.A. On the Formation of the Acid Sites in Lanthanum Exchanged X Zeolites Used for Isobutane/Cis-2-Butene Alkylation. *Microporous and Mesoporous Materials* **2005**, *83*, 309–318, doi:10.1016/j.micromeso.2005.04.024.
13. Yassin, F.A.; El Kady, F.Y.; Ahmed, H.S.; Mohamed, L.K.; Shaban, S.A.; Elfadaly, A.K. Highly Effective Ionic Liquids for Biodiesel Production from Waste Vegetable Oils. *Egyptian Journal of Petroleum* **2015**, *24*, 103–111, doi:10.1016/j.ejpe.2015.02.011.
14. Capeletti, L.B.; Zimnoch, J.H. Fourier Transform Infrared and Raman Characterization of Silica-Based Materials. *Applications of Molecular Spectroscopy to Current Research in the Chemical and Biological Sciences* **2016**, doi:10.5772/64477.
15. Scardera, G.; Puzzer, T.; Conibeer, G.; Green, M.A. Fourier Transform Infrared Spectroscopy of Annealed Silicon-Rich Silicon Nitride Thin Films. *Journal of Applied Physics* **2008**, *104*, 104310, doi:10.1063/1.3021158.
16. Müller, M.; Villalba, J.C.; Anaissi, F.J. Thermal Decomposition (TG-DTA) of Iron Salts [$FeCl_3.6H_2O$] and [$Fe(NO_3)_3.9H_2O$] with Morphologic and Chemical Analysis of Final Product. *Semina: Ciências Exatas e Tecnológicas* **2014**, *35*, 9–14, doi:10.5433/1679-0375.2014v35n1p9.
17. Kingery, W.D.; Uhlmann, D.R.; Bowen, H.K. *Introduction to Ceramics*; 2nd ed.; New York : Wiley, 1976; ISBN 978-0-471-47860-7.
18. Yoon, S.-Y.; Lee, C.-G.; Park, J.-A.; Kim, J.-H.; Kim, S.-B.; Lee, S.-H.; Choi, J.-W. Kinetic, Equilibrium and Thermodynamic Studies for Phosphate Adsorption to Magnetic Iron Oxide Nanoparticles. *Chemical Engineering Journal* **2014**, *236*, 341–347, doi:10.1016/j.cej.2013.09.053.
19. Zeng, L.; Li, X.; Liu, J. Adsorptive Removal of Phosphate from Aqueous Solutions Using Iron Oxide Tailings. *Water Research* **2004**, *38*, 1318–1326, doi:10.1016/j.watres.2003.12.009.
20. Lalley, J.; Han, C.; Li, X.; Dionysiou, D.D.; Nadagouda, M.N. Phosphate Adsorption Using Modified Iron Oxide-Based Sorbents in Lake Water: Kinetics, Equilibrium, and Column Tests. *Chemical Engineering Journal* **2016**, *284*, 1386–1396, doi:10.1016/j.cej.2015.08.114.
21. Shahid, M.K.; Kim, Y.; Choi, Y.-G. Magnetite Synthesis Using Iron Oxide Waste and Its Application for Phosphate Adsorption with Column and Batch Reactors. *Chemical Engineering Research and Design* **2019**, *148*, 169–179, doi:10.1016/j.cherd.2019.06.001.
22. Cao, D.; Jin, X.; Gan, L.; Wang, T.; Chen, Z. Removal of Phosphate Using Iron Oxide Nanoparticles Synthesized by Eucalyptus Leaf Extract in the Presence of CTAB Surfactant. *Chemosphere* **2016**, *159*, 23–31, doi:10.1016/j.chemosphere.2016.05.080.
23. Ajmal, Z.; Muhmood, A.; Usman, M.; Kizito, S.; Lu, J.; Dong, R.; Wu, S. Phosphate Removal from Aqueous Solution Using Iron Oxides: Adsorption, Desorption and Regeneration Characteristics. *Journal of Colloid and Interface Science* **2018**, *528*, 145–155, doi:10.1016/j.jcis.2018.05.084.

24. Daou, T.J.; Begin-Colin, S.; Grenèche, J.M.; Thomas, F.; Derory, A.; Bernhardt, P.; Legaré, P.; Pourroy, G. Phosphate Adsorption Properties of Magnetite-Based Nanoparticles. *Chem. Mater.* **2007**, *19*, 4494–4505, doi:10.1021/cm071046v.
25. Li, M.; Liu, H.; Chen, T.; Wei, L.; Wang, C.; Hu, W.; Wang, H. The Transformation of α-(Al, Fe)OOH in Natural Fire: Effect of Al Substitution Amount on Fixation of Phosphate. *Chemical Geology* **2019**, *524*, 368–382, doi:10.1016/j.chemgeo.2019.07.008.
26. Sousa, A.F. de; Braga, T.P.; Gomes, E.C.C.; Valentini, A.; Longhinotti, E. Adsorption of Phosphate Using Mesoporous Spheres Containing Iron and Aluminum Oxide. *Chemical Engineering Journal* **2012**, *210*, 143–149, doi:10.1016/j.cej.2012.08.080.
27. Xu, J.; Luu, L.; Tang, Y. Phosphate Removal Using Aluminum-Doped Magnetic Nanoparticles. *Desalination and water treatment* **2017**, *58*, doi:10.5004/dwt.2017.0356.
28. Mezenner, N.Y.; Bensmaili, A. Kinetics and Thermodynamic Study of Phosphate Adsorption on Iron Hydroxide-Eggshell Waste. *Chemical Engineering Journal* **2009**, *147*, 87–96, doi:10.1016/j.cej.2008.06.024.
